# Assessing variability in breast cancer management across the world: results of a questionnaire survey amongst global international experts in breast cancer management

**DOI:** 10.3332/ecancer.2022.1443

**Published:** 2022-09-02

**Authors:** Dinesh Thekkinkattil, Raghavan Vidya, Ava Kwong, Adil Aljarrah Alajmi, Miriam Mutebi, Bahadir Gulluoglu, Suryanarayana Deo, Eisuke Fukuma, Elisabeth Elder, Eduardo Gonzalez, Fredrik Warnberg, Iness Buccimazza, Owen Ung, Melanie Walker, Maria Vernet-Tomas, Marie-Jeanne TFD Vrancken Peeters, Nathalie Johnson, Regis Resende Paulinelli, Thorsten Kuehn, Paolo Veronesi, Diptendra Sarkar, Jill Dietz

**Affiliations:** 1 Lincoln Breast Unit, Lincoln County Hospital, United Lincolnshire Hospitals NHS Trust, Lincoln LN2 5QY, U.K.; 2 The Royal Wolverhampton Hospital NHS Trust, Wolverhampton WV10 0QP, U.K.; 3 Department of Surgery, Queen Mary and Tung Wah Hospital and The University of Hong Kong-ShenZhen Hospital, China; 4 Breast Program, Sultan Qaboos Comprehensive Cancer Care and Research Centre (SQCCCRC), Muscat, Sultanate of Oman; 5 Department of Surgery, Aga Khan University Hospital, Nairobi, Kenya; 6 Department of General Surgery, Marmara University School of Medicine, Istanbul, Turkey; 7 Department of Surgical Oncology, All India Institute of Medical Sciences, New Delhi 11002, India; 8 Breast Center, Kameda Medical Center, Japan; 9 Westmead Breast Cancer Institute, Westmead Hospital, University of Sydney, NSW 2145, Australia; 10 Department of Surgical Oncology, Institute of Oncology ‘Ángel H Roffo’, Av San Martín 5481, Universidad de Buenos Aires, Buenos Aires, C1417 CABA, Argentina; 11 Department of Surgery, Sahlgrenska Academy, Gothenburg University, Sweden; 12 Breast and Endocrine Unit, Department of Surgery, Nelson R. Mandela School of Medicine, University of KwaZulu-Natal, Durban, South Africa; 13 MNHHS Comprehensive Breast Cancer Institute, Royal Brisbane and Women’s Hospital, University of Queensland, Herston, Qld 4029, Australia; 14 Department of Surgery, The Alfred Hospital, Prahran, Victoria, 3181, Australia; 15 Breast Diseases Unit, Hospital del Mar, Parc de Salut Mar, 08003 Barcelona, Spain; 16 Department of Surgery, Antoni van Leeuwenhoek, Plesmanlaan 121, 1066 CX Amsterdam, The Netherlands; 17 Department of Surgery, Amsterdam University Medical Center, Amsterdam, The Netherlands; 18 Legacy Cancer Institute, Portland, Oregon, USA; 19 Mastology Program, Federal University of Goias, Brazil; 20 Araujo Jorge Cancer Hospital, Goias Anti-Cancer Association, Brazil; 21 Interdisciplinary Breast Center, Klinikum Esslingen, Germany; 22 Department of Breast Surgery, IRCCS Istituto Europeo di Oncologia, Via Ripamonti 435, 20141 Milano, Italy; 23 University of Milan, School of Medicine, Italy; 24 Breast Service, Department of Surgery, Institute of Post-Graduate Medical Education and Research, AJC Bose Rd, Kolkata, India; 25 Allegheny Health Network Cancer Institute, Pittsburgh, PA 15212, USA

**Keywords:** breast cancer, global health, questionnaire, socioeconomic status, training

## Abstract

**Background:**

Breast cancer is the most common cancer in women worldwide with an estimated 2.3 million breast cancer cases diagnosed annually. The outcome of breast cancer management varies widely across the globe which could be due to a multitude of factors. Hence, a blanket approach in standardisation of care across the world is neither practical nor feasible.

**Aim:**

To assess the extent and type of variability in breast cancer management across the globe and to do a gap analysis of patient care pathway.

**Method:**

An online questionnaire survey and virtual consensus meeting was carried out amongst 31 experts from 25 countries in the field of breast cancer surgical management. The questionnaire was designed to understand the variability in diagnosis and treatment of breast cancer, and potential factors contributing to this heterogeneity.

**Result:**

The questionnaire survey shows a wide variation in breast surgical training, diagnosis and treatment pathways for breast cancer patients. There are several factors such as socioeconomic status, patient culture and preferences, lack of national screening programmes and training, and paucity of resources, which are barriers to the consistent delivery of high-quality care in different parts of the world.

**Conclusion:**

On-line survey platforms distributed to global experts in breast cancer care can assess gaps in the diagnosis and treatment of breast cancer patients. This survey confirms the need for an in-depth gap analysis of patient care pathways and treatments to enable the development of personalised plans and policies to standardise high quality care.

## Background

Incidence and mortality associated with breast cancer varies widely across the globe. There are several socioeconomic and population-based factors which contribute to this heterogeneity [1, 2]. There is a wide variation in the way the breast cancer is identified and managed in different parts of the world [3]. Global Breast Hub is a non-charitable, non-profit organisation set up to establish correspondence to promote, standardise and advance safe and effective breast care around the globe. It could be the factors such as availability of resources, cost of treatment, availability of adequate training, availability of needed skill, variation in culture playing a role for the disparity and variability in breast cancer management across the globe. There is a pressing need to identify the knowledge gap in various aspects of breast cancer diagnosis and management across the globe. This will help to draft personalised policies and solutions to provide high-quality standardised care for our breast cancer patients across the world.

## Aim

To assess variability in breast cancer management across the globe and to do the gap analysis of patient care pathway.

## Methods

**Figure 1.**
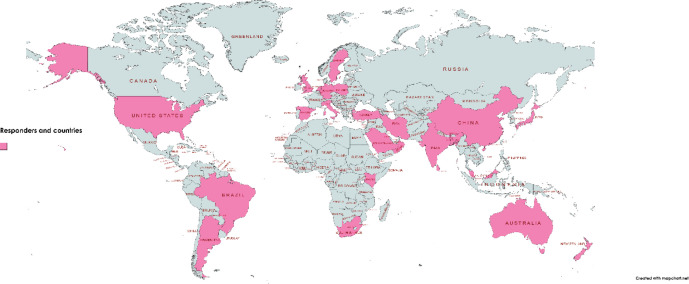
Responders and countries.

The initial members of Global Breast Hub organised a group of Prime members who are leading experts in Breast Surgery with the ability to reach most of the surgeons caring for breast cancer patients in their respective countries. An online questionnaire survey was distributed amongst 31 experts from 25 countries (Figure 1). This Questionnaire was designed to identify global gaps in care and establish ability of our members to reach practicing surgeons in their country. Questionnaire survey had 41 components assessing the various aspects of diagnosis, multidisciplinary management and barriers in training and treatment of breast cancer management (Appendix). A follow-up consensus virtual meeting was held to review and analyse the results of the survey.

## Results

**Figure 2.**
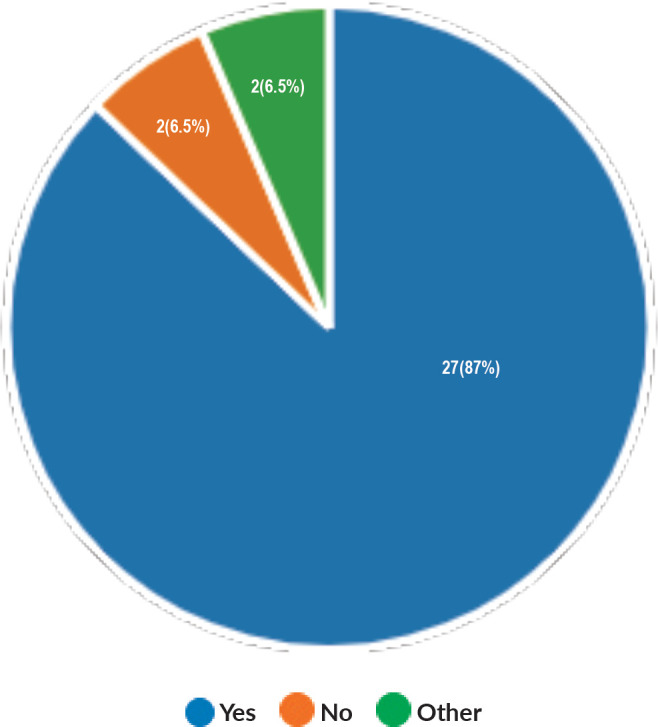
Availability of National Health Care System.

**Figure 3.**
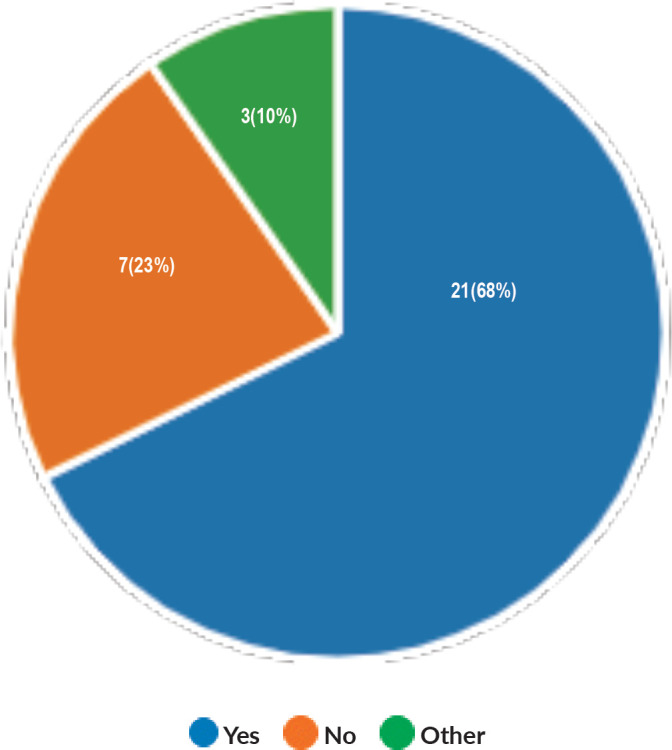
Availability of National Cancer Registry.

**Figure 4.**
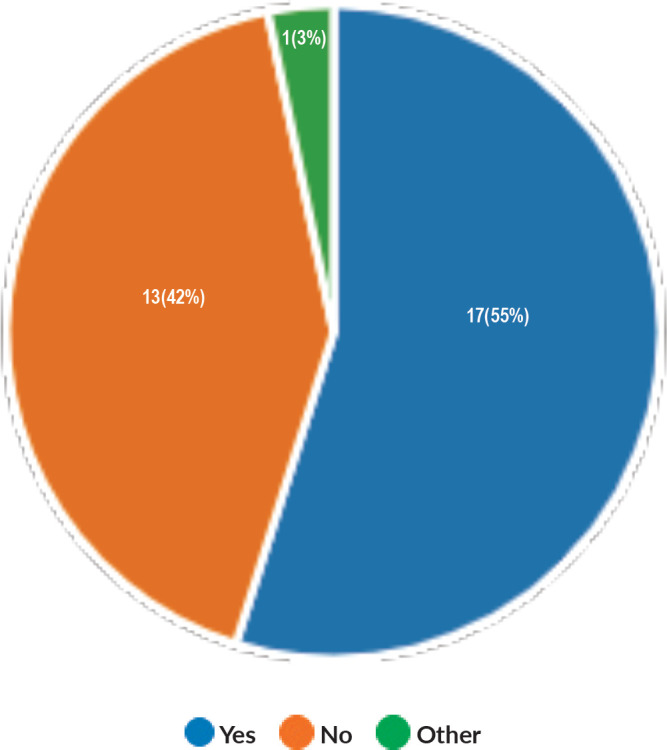
Availability of National Breast Screening Programme.

**Figure 5.**
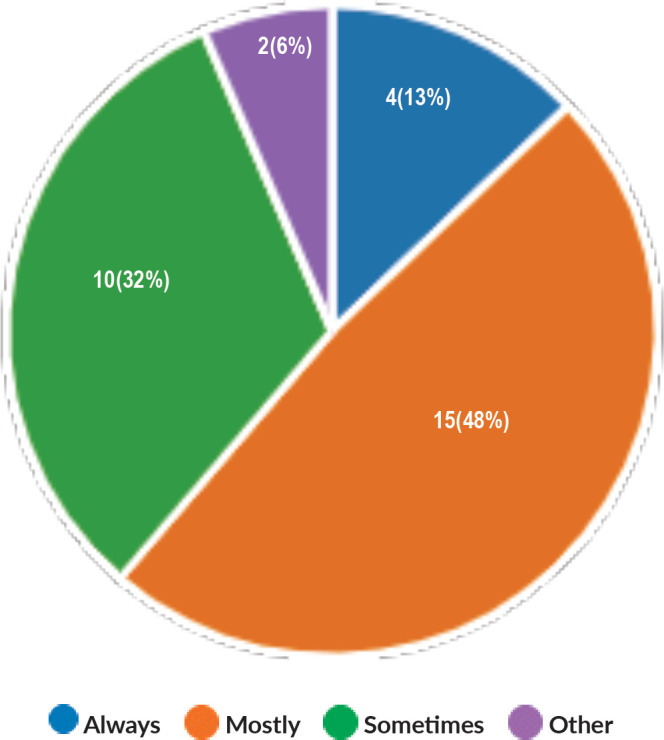
Breast cancer management in dedicated or accredited breast centres with multidisciplinary care team.

**Figure 6.**
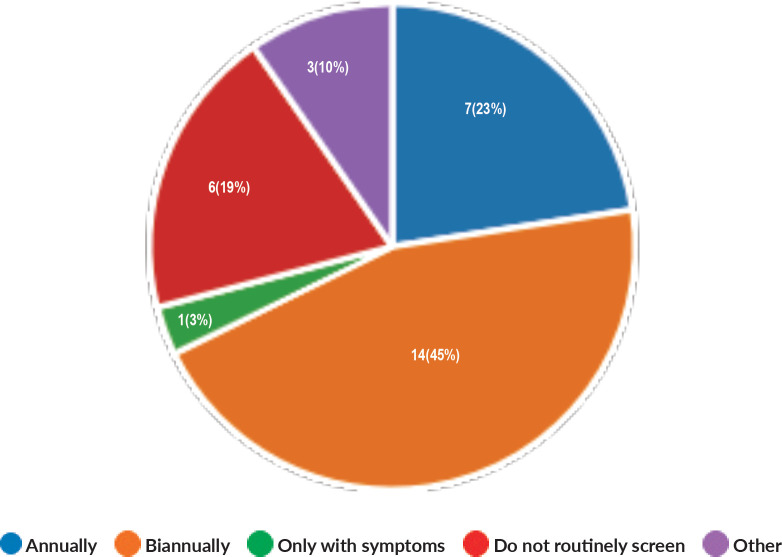
Frequency of breast screening.

**Figure 7.**
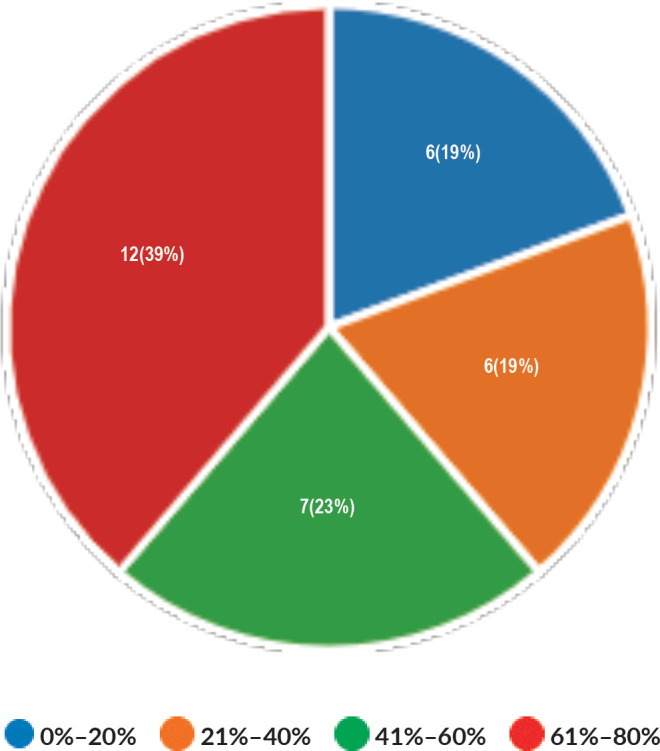
Proportion of patient undergoing breast conservation surgery.

**Figure 8.**
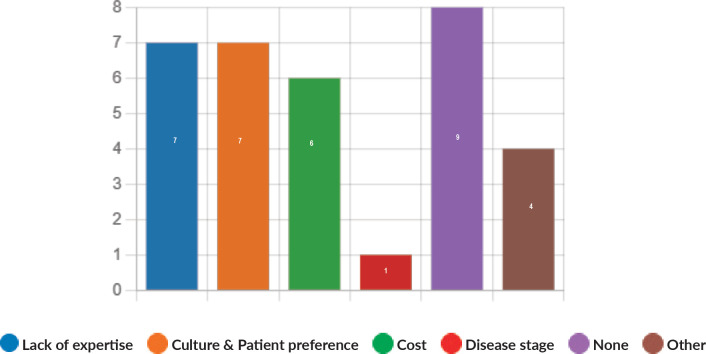
Most important hurdles in offering breast reconstruction, if any.

**Figure 9.**
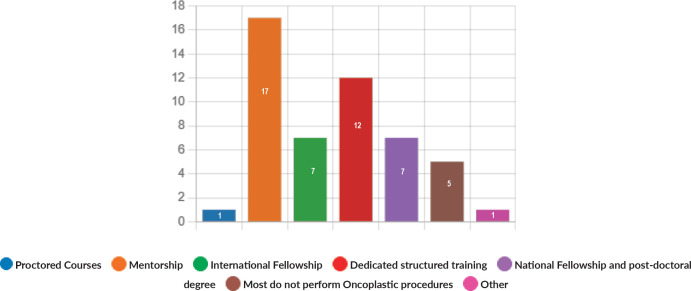
Mode of training in oncoplastic procedures.

The main results from the questionnaire were as follows:

Majority of the members confirmed that they had a National Health Care System in their country (87%) and 68% had a National Cancer Registry (Figures 2 and 3). Only 55% members confirmed that their country had a national breast screening programme (Figure 4). Only a minority confirmed that there was limited access to dedicated breast centres to manage breast cancer and 61% panellists confirmed that breast cancer is mostly managed in dedicated breast centres (Figure 5). Significant variability was noted regarding access to a national breast screening programme and the age and frequency at which breast screening is offered across the globe: 45% respondents confirmed the age at which screening starts is between 40 and 50 years whilst 32% confirmed that the screening starts over the age of 50 years. Likewise, 45% of our experts confirmed that the frequency of screening is biannually whereas 23% offered screening every year and 3% of panellists suggested that the screening is carried out every 3 years and 19% panellists said that they do not have a routine breast screening programme in their country (Figure 6). Digital mammography was the most used modality for screening programme (68%). Significant proportion of members confirmed that the breast cancer surgery was carried out by dedicated breast surgeons (49%). General surgeons (29%), gynaecologists (8%) and surgical oncologist (14%) were also offering surgery for breast cancer patients. 65% members confirmed that the breast surgery decision was discussed by a multidisciplinary team or tumour board and in 32% responses, this decision was made by the treating breast surgeons. Similarly, medical oncology treatment decisions were made by a Multidisciplinary Team/Tumour board in 51% and in 37% members reported that these decisions were made by the treating medical oncologist. Majority (48%) confirmed that they follow their own national guidelines for treatment guidance and almost all members confirmed that they use various national and international guidelines to guide their treatment protocol. There was significant variability in the practice of breast conservation surgery (Figure 7). The percentage of patients undergoing breast conserving surgery varied from 1% to 80%. Advanced stage of disease presentation was ranked as the major obstacle for offering breast conservation surgery (25%). Other barriers for breast conservation surgery were as follows: patient choice and compliance (20%), lack of expertise (14%) and lack of availability of radiotherapy (17%). Similarly, there was significant variation in availability of immediate breast reconstruction in different parts of the world. 6% responses confirmed lack of availability in their country, whereas 29% responses confirmed less than 10% of their patients having immediate breast reconstruction after mastectomy. 45% of respondents confirmed that over 20% of their mastectomy patients undergo immediate breast reconstruction. The proportion of patients having delayed breast reconstruction was also similarly heterogenous: 19% respondents suggested that delayed reconstruction option is not available for patients having mastectomy, 71% of the experts confirmed that 10%–20% of their patients having mastectomy had delayed reconstruction in their country and a smaller proportion (19%) of panellists confirmed that over 20% of their mastectomy patients are having delayed reconstruction. 52% members confirmed that a range of autologous and implant-based reconstructions were available in their country. Majority of respondents reported that breast reconstructions are conducted by plastic surgeons (48%) and dedicated oncoplastic breast surgeons (48%) or as combined approach by breast and plastic surgeons (39%). Majority of respondents confirmed availability of government funding for reconstruction (52%) and 39% responses suggested funding by insurance companies. 42% experts reported that the reconstructions were funded by patients. Cost, lack of expertise, culture and patient preferences were ranked as the major barriers to offering breast reconstruction (Figure 8). There was no specialised licensing examination for breast surgery in majority (71%) of countries taken part in the survey; however, national or regional fellowships dedicated for breast cancer surgical management was available according to 61%. Combination of structured training, national and international fellowships and mentorships was used for oncoplastic surgical training (Figure 9). 94% respondents confirmed availability of sentinel lymph node procedure and all respondents were carrying out percutaneous needle biopsy for diagnosis. Only a small percentage of respondents confirmed that Radiotherapy was not used or feasible after breast conservation surgery (6%). 84% confirmed use of genomic testing in their practice. 94% respondents confirmed the use of targeted therapy for HER 2 positive cancers. 96.7% of responses confirmed that estrogen receptor (ER), progesterone receptor (PR) and HER 2 markers are routinely performed on biopsy. Respondents identified the following factors as barrier for excellent and consistent care for breast cancer patients: Surgery being done by low volume surgeons (45%), lack of unified multi-disciplinary team (39%), lack of adequate funding or resources (35%) and lack of central body or oversight (23%)

## Discussion

Globally the incidence, variability in treatment and financial burden of breast cancer is rising [4, 5]. Participants in our survey, experts in the field of breast cancer management from 25 countries with variable socioeconomic background, participated in the virtual survey and conference and showed active engagement in the mission of the organisation. Most of the members hold key positions in national or international associations involved with quality improvement and setting standards in breast cancer management. The Global Breast Hub survey platform, through these prime members and the involvement of other key stakeholders across the globe, can successfully identify gaps in diagnosis and treatment. The hurdles and obstacles for delivery of consistent and excellent standard of care for breast cancer patients differ depending on the region [6]. Several factors such as funding and resources, cultural differences and basic training structure do play a major role in this heterogeneity of care [7, 8]. This stresses the importance of inadequacy of a blanket approach addressing the standard of care globally and need for individualised approach aiming specific needs in different parts of the world.

Our survey supports the literature that breast cancer screening availability varies globally as does technology available for screening [9]. We identified that late-stage diagnosis as a barrier to standard of care treatment. Our survey suggests that there are deficiencies in structured cancer diagnostic and management pathway in many low and middle income countries which could be a major hurdle for achieving optimal patient outcome.

The questionnaire survey confirms a wide variation in both training and education as well as treatment protocols followed in different parts of the world. Availability of speciality breast training including oncoplastic breast surgery remains heterogenous and requires streamlining and closer collaboration globally [10]. The use of structured training, targeted curriculum and quality assurance is essential to address global delivery of high quality of breast service [11].

Global breast hub, using this survey platform, aims to identify global gaps in breast cancer diagnosis and treatment and work toward eliminating disparities in breast care by partnering with other global stakeholders whose mission is to improve education, allocate resources and change policy.

## Conclusion

Our survey amongst experts in the field of breast cancer surgery from six continents confirms significant variation in the availability of resources to diagnose and treat breast cancer across the globe. In addition, the survey confirms a lack of availability of structured training and a lack of expertise in some parts of the world. Our survey demonstrates the need for a tailored approach to address the knowledge gap and resource availability across the world to optimise breast cancer management and maximise survival and treatment outcomes.

## Source of funding

None.

## Conflicts of interest

None to declare from any authors.

## Appendix. Questionnaire used in the survey.

1. **What is your title?**
  - Consultant
  - Attending
  - Professor/Associate Professor/Assistant Professor
  - _
2. **How many years in practice?**
3. **Choose the top 3 interests to discuss in Global Breast Hub:**
  - Education/Training
  - Research
  - Treatment dilemmas
  - Global and environmental impact on breast disease (pandemic, obesity, smoking, etc.)
  - Health care inequalities/disparities
  - Quality metrics
  - New devices
  - New techniques
  - Healthcare management
4. **Organisational membership:**
  - ASBrS
  - ABS
  - ABSI
  - SSO
  - ESSO
  - Breast Surgery International (BSI)
  - _
5. **Role in organisational membership:**
  - Member
  - President
  - Past President
  - Secretary
  - Treasurer
  - Chair of Committee
  - _
6. **Name of country where you live and practice**
7. **Is the number of breast cancers in your country in the last decade**
  - Increasing
  - Decreasing
  - Stable
8. **Does your country have a National Health Care System?**
  - Yes
  - No
  - _
9. **Does your country have a National Cancer Registry?**
  - Yes
  - No
  - _
10. **If your country has a National Cancer Registry, what percentage of the cancer breast population uses the data base?**
11. **Are most of the breast cancer patients in your country managed in dedicated or accredited breast centres with a multidisciplinary care team?**
  - Always
  - Mostly
  - Sometimes
  - Never
  - _
12. **Does your country have a National Breast Screening Programme?**
  - Yes
  - No
  - _
13. **If your country has a National Breast Screening Programme, when was it initiated?**
  - 2010 onwards
  - 2000–2009
  - 1990–1999
  - 1980–1989
  - before 1980
14. **What age do you start image screening in your country?**
  - <40 years
  - 40–50 years
  - >50 years
  - Do not routinely screen
15. **What frequency do you screen in your country?**
  - Annually
  - Biannually
  - Only with symptoms
  - Do not routinely screen
  - _
16. **What is the most common screening modality in your country?**
  - Digital mammography
  - Digital tomosynthesis
  - Ultrasound
  - Clinical breast examination
  - Analogue mammography
  - MRI
  - No screening
17. **Who performs majority of breast cancer surgery in your country? *check in order**
  - General Surgeon
  - Breast Surgeon
  - Gynaecologist
  - Surgical Oncologist
18. **Who decides surgical treatment of breast cancer?**
  - MDT/Tumour Board
  - Treating Surgeon
  - _
19. **Who decides medical oncological treatment of Breast Cancer?**
  - MDT/Tumour Board
  - Oncologist
  - Treating Surgeon
  - _
20. **What type of MDT (Multidisciplinary/Tumour Board) do you have in your country?**
  - Virtual
  - Face to face meeting with all core members
  - Hybrid
  - Do not have an MDT/Tumour Board
  - _
21. **Which treatment guidelines do you follow for treating breast cancer patients?**
  - NCCN
  - ASCO
  - ESMO
  - St. Gallen Consensus
  - Own National guidelines (ASBrs/ABS/ESSO/SSO/ABSI)
  - No guidelines
  - _
22. **What proportion of breast cancer patients undergo Breast Conservation Surgery in your Country?**
  - 0%–20%
  - 21%–40%
  - 41%–60%
  - 61%–80%
  - 81%–100%
23. **What is the main obstacle in practicing Breast Conservation Surgery in your country?**
  - Patient choice and compliance issues with radiotherapy
  - Advanced state of breast cancer at presentation
  - Expertise not available
  - No obstacles
  - Lack of availability of radiotherapy facility
  - _
24. **What percentage of patients undergoing mastectomy undergo immediate Breast Reconstruction in your country?**
  - 1%–10%
  - 10%–20%
  - 20%–40%
  - 40%–60%
  - 60%–80%
  - 80%–100%
  - Not available
25. **What percentage of patients undergoing mastectomy undergo delayed Breast Reconstruction in your country?**
  - Not Available
  - 1%–10%
  - 10%–20%
  - 20%–40%
  - 40%–60%
  - 60%–80%
  - 80%–100%
26. **What type of breast reconstruction is routinely offered in your country?**
  - All types of reconstructions
  - Implant based
  - Autologous tissue based: pedicled and free flaps
  - Autologous tissue based: only pedicled flaps
  - None
  - _
27. **Who performs breast reconstruction in your country?**
  - Plastic Surgeon
  - Breast Surgeon
  - Oncoplastic Breast Surgeon
  - Combined Procedure by Breast Surgeon and Plastic Surgeon
  - Not done
  - _
28. **Who funds breast reconstruction procedures in your country?**
  - Government
  - Covered by Insurance Company
  - Self-funded by patients
  - _
29. **What are the most important hurdles in offering breast reconstruction in your country, if any?**
  - Lack of expertise
  - Culture and patient preferences
  - Cost
  - Disease stage
  - None
  - _
30. **Does a special licence exam exist for breast surgery in the country?**
  - Yes
  - No
31. **Are there National or Regional fellowships available dedicated for breast cancer surgical management?**
  - Yes
  - No
32. **How do you get trained in Oncoplastic procedures in your country?**
  - Proctored courses
  - Mentorship
  - International fellowships
  - Dedicated structured training program
  - National fellowships and post-doctoral degree
  - Most Do not perform oncoplastic procedures
  - _
33. **Do you offer Sentinel Lymph node biopsy for most early- stage breast cancer patients in your country?**
  - Yes
  - No
  - _
34. **Do you perform percutaneous needle biopsy for diagnosis of most breast cancers in your country?**
  - Yes
  - No
35. **Is breast irradiation feasible and utilised for most patients undergoing breast conservation or if node positive?**
  - Yes
  - No
36. **Do you use genomic testing in your country?**
  - Yes
  - No
37. **If you use genomic testing, what percentage done on the core?**
38. **Do you have targeted therapy for the treatment of HER 2+ breast cancer in your country?**
  - Yes
  - No
39. **What markers do you obtain from pathology? Mark all that apply.**
  - ER
  - PR
  - HER 2
  - Ki 67
  - Oncotype
  - _
40. **Do you have a mechanism or ability to contact most of the treating surgeons in your country?**
  - Yes
  - No
  - _
41. **What is the largest barrier to excellent and consistent care for all breast cancer patients in your country? Check all that apply.**
  - Surgery being done by surgeons who do few breast cases per year
  - Lack of unified multidisciplinary teams
  - No accrediting bodies or central oversight
  - Access to radiation facilities
  - Access to medical therapies
  - None
  - Lack of funding or resources
  - _
